# Target of Rapamycin Is a Key Player for Auxin Signaling Transduction in *Arabidopsis*

**DOI:** 10.3389/fpls.2016.00291

**Published:** 2016-03-11

**Authors:** Kexuan Deng, Lihua Yu, Xianzhe Zheng, Kang Zhang, Wanjing Wang, Pan Dong, Jiankui Zhang, Maozhi Ren

**Affiliations:** ^1^School of Life Sciences, Chongqing UniversityChongqing, China; ^2^College of Agronomy and Biotechnology, Southwest UniversityChongqing, China

**Keywords:** target of rapamycin, FKBP12, rapamycin, KU63794, auxin

## Abstract

Target of rapamycin (TOR), a master sensor for growth factors and nutrition availability in eukaryotic species, is a specific target protein of rapamycin. Rapamycin inhibits TOR kinase activity viaFK506 binding protein 12 kDa (FKBP12) in all examined heterotrophic eukaryotic organisms. In *Arabidopsis*, several independent studies have shown that AtFKBP12 is non-functional under aerobic condition, but one study suggests that AtFKBP12 is functional during anaerobic growth. However, the functions of AtFKBP12 have never been examined in parallel under aerobic and anaerobic growth conditions so far. To this end, we cloned the *FKBP12* gene of humans, yeast, and *Arabidopsis*, respectively. Transgenic plants were generated, and pharmacological examinations were performed in parallel with *Arabidopsis* under aerobic and anaerobic conditions. ScFKBP12 conferred plants with the strongest sensitivity to rapamycin, followed by HsFKBP12, whereas AtFKBP12 failed to generate rapamycin sensitivity under aerobic condition. Upon submergence, yeast and human FKBP12 can significantly block cotyledon greening while *Arabidopsis* FKBP12 only retards plant growth in the presence of rapamycin, suggesting that hypoxia stress could partially restore the functions of AtFKBP12 to bridge the interaction between rapamycin and TOR. To further determine if communication between TOR and auxin signaling exists in plants, yeast *FKBP12* was introduced into *DR5::GUS* homozygous plants. The transgenic plants DR5/BP12 were then treated with rapamycin or KU63794 (a new inhibitor of TOR). GUS staining showed that the auxin content of root tips decreased compared to the control. DR5/BP12 plants lost sensitivity to auxin after treatment with rapamycin. Auxin-defective phenotypes, including short primary roots, fewer lateral roots, and loss of gravitropism, occurred in DR5/BP12 plants when seedlings were treated with rapamycin+KU63794. This indicated that the combination of rapamycin and KU63794 can significantly inhibit TOR and auxin signaling in DR5/BP12 plants. These studies demonstrate that TOR is essential for auxin signaling transduction in *Arabidopsis*.

## Introduction

Rapamycin (also known as sirolimus), a macrolide antibiotic, is produced by the soil bacterium, *Streptomyces hydroscopicus*. All tested heterotrophic eukaryotes, including yeast, nematodes (*Caenorhabditiselegans*), fruit flies (*Drosophila*), and mammals, are sensitive to rapamycin (Xiong and Sheen, 2015). Rapamycin feeding mimics nutrition restriction and energy depletion and thus significantly extends the lifespans of mammals and plants (Fontana et al., 2010; Ren et al., 2012). Rapamycin is therefore a research focus of human preventive medicine. Interestingly, most plants are insensitive to rapamycin under aerobic growth condition (Xu et al., 1998; Menand et al., 2002; Ren et al., 2012; Montané and Menand, 2013). This insensitivity might involve self-immune mechanisms. From an evolutionary perspective, billions of years separate the beginnings of yeast and the development of human cells, yet human cell exposure to rapamycin produces the same nutrition starvation phenomenon as found in yeast. This indicates a highly conserved target for rapamycin in eukaryotes. *TOR1* and *TOR2* genes, targets of rapamycin, have been identified in budding yeast and this has allowed advanced TOR studies (Cafferkey et al., 1993; Kunz et al., 1993; Sabatini et al., 1994; Chen et al., 1995; Loewith et al., 2002). Since its initial discovery, the *TOR* gene has been isolated from all examined eukaryotic organisms. Most eukaryotic organisms contain only one *TOR* gene, whereas two and three *TOR* genes exist in yeast and *Leishmania major*, respectively (Madeira Da Silva and Beverley, 2010). Disruption of the *TOR* gene is lethal in eukaryotes, indicating that TOR is required for life in eukaryotic cells (Wullschleger et al., 2006). Disruption of the TOR signal is one of the major causes of nutrition-related diseases in animals and humans, including diabetes, cancer, and cardiovascular disease (Zagouri et al., 2012; Cornu et al., 2013). TOR function is highly conserved from yeast to humans, and it controls key biological processes such as ribosome biogenesis, protein synthesis, three carboxylic acid cycles, and stress responses (Fontana et al., 2010; Cornu et al., 2013).

The 12-KDa FK506-binding protein 12 (FKBP12) is the receptor protein of rapamycin and it mediates the interaction between TOR and rapamycin (Brown et al., 1994). In yeast and mammals, rapamycin first forms a heterogeneous complex with FKBP12 and then specifically targets and binds to the FRB domain of TOR to form a rapamycin-FKBP12-TOR complex that in turn inhibits the kinase activity of TOR (Chiu et al., 1994; Sabatini et al., 1994; Choi et al., 1996). In this rapamycin-FKBP12-TOR system, FKBP12 plays a crucial role by being directly involved in rapamycin recognition and binding. Mutations in the *FKBP12* gene result in rapamycin insensitivity in yeast (Koltin et al., 1991). TOR deletion is lethal, and all tested fungi and animals are sensitive to rapamycin (Heitman et al., 1991; Loewith et al., 2002; Wullschleger et al., 2006). Based on the rapamycin-FKBP12 negative regulation system of TOR, TOR and its signaling pathway in yeast and animals have been extensively studied. The structure and function of FKBP12 proteins are highly conserved and human FKBP12 can functionally complement that of yeast (Koltin et al., 1991). Although the amino acid sequences of plant FKBP12s are relatively similar to those of yeast and mammals, wild-type (WT) *Arabidopsis* is insensitive to rapamycin and does not express any detectable phenotypes even at high concentrations (20 μg/mL rapamycin) under aerobic condition (Xu et al., 1998; Menand et al., 2002; Mahfouz et al., 2006; Sormani et al., 2007; Ren et al., 2012; Montané and Menand, 2013). Various genetic, biochemical, yeast two hybrid (Y2H), and pharmacological analyses have demonstrated that plant FKBP12 does not have the ability to form rapamycin/FKBP12/TOR complexes (Xu et al., 1998; Sormani et al., 2007; Moreau et al., 2012; Ren et al., 2012; Montané and Menand, 2013). However, overexpression of *AtFKBP12* can dramatically enhance rapamycin sensitivity during anaerobic growth (Xiong and Sheen, 2012). This observation invalidates the common belief that TOR signaling in *Arabidopsis* is always insensitive to rapamycin, but the AtFKBP12 transgenic plants have never been carefully examined in parallel under aerobic and anaerobic growth condition so far.

Growth factors such as insulin and IGF in mammals are the key signals determining cell growth, proliferation, differentiation, and fate. TOR has a close relationship with growth factors (Wang et al., 2006; Vander Haar et al., 2007; Feng and Levine, 2010). In plants, auxin is the major phytohormone and growth factor controlling the cell cycle, division, elongation, differentiation, growth, and development (Teale et al., 2006). Auxin and growth factors can activate the TOR signaling pathway to modulate specific mRNA transcription, translation, translation re-initiation, and selective protein synthesis by phosphorylating key downstream regulators such as S6 ribosomal protein kinase (S6K), S6 ribosomal protein, and eukaryotic initiation factor 4E (eIF4E; Dinkova et al., 2000; Beltrán-Peña et al., 2002; Bögre et al., 2013; Schepetilnikov et al., 2013; Villa-Hernández et al., 2013). Our recent observations indicate that many auxin signaling associated genes are differentially expressed in *Arabidopsis* seedlings treated with AZD8055 (one of asTORis; Dong et al., 2015). TOR therefore appears to play a crucial role in mediating auxin signals. However, one study reported that auxin is decoupled from TOR activation in *Arabidopsis* (Xiong et al., 2013). To clarify the issues concerning FKBP12 functions and the relationship between auxin and TOR signaling in plants, we cloned *FKBP12* from human (*HsFKBP12*), yeast (*ScFKBP12*) and *Arabidopsis* (*AtFKBP12*) and generated overexpression transgenic lines of *Arabidopsis*. The functions of HsFKBP12, ScFKBP12 and AtFKBP12 were tested in parallel for their sensitivity to rapamycin under aerobic and anaerobic conditions. Our results demonstrate that plants can acquire rapamycin sensitivity by overexpression of HsFKBP12 or ScFKBP12, and ScFKBP12 showed more pronounced effects than HsFKBP12 under both aerobic and anaerobic growth conditions. No rapamycin response was observed from transgenic plants containing *P35S::AtFKBP12* during aerobic growth, but the rapamycin sensitivity can be partially rescued in response to submergence. Double transgenic plants named after DR5/BP12s, which contained *P35S::ScFKBP12* and *DR5:: GUS*, were generated. When DR5/BP12 plants were simultaneously treated with rapamycin and KU63794 (KU), normal growth and development of the roots was inhibited and the root geotropism was also altered. This was a classic phenotype of defective auxin signaling. GUS staining showed that auxin distribution was reduced in DR5/BP12 plants in the presence of the TOR inhibitor. TOR significantly influenced the concentration and response of auxin in the plant and altered the downstream signal response processes.

## Materials and methods

### Plant materials and growth conditions

In the present study, wild-type (WT) *Arabidopsis* Columbia (Columbia-0) ecotype and transgenic lines were grown in growth chambers with 16 h light/8 h dark photoperiod at 22°C. Transgenic plants were generated using the floral dipping method (Zhang et al., 2006). In all cases, *A. thali*ana seeds were surface-sterilized for 5 min in 70% ethanol, and then washed in 10% sodium hypochlorite containing 0.3% Tween-20. Finally, seeds were washed five times with sterilized water. Before sowing on plates, the seeds were incubated for 2 days at 4°C in the dark.

### RNA extraction and generation of overexpression constructs

Total RNA was extracted from *Arabidopsis* Columbia (Columbia-0) and human cells using TRIzol (Invitrogen), following the manufacturer's protocol. cDNA was synthesized by using the TransScript One-Step gDNA Removal and cDNA Synthesis SuperMix kit (Transgen). The full-length coding sequence of *HsFKBP12* and *AtFKBP12* was amplified by TransStarTaq DNA Polymerase (Transgen) using the corresponding primers (Supplementary Table 1). PCR primers for amplification of the full-length genes from *Arabidopsis* were designed based on its cDNA sequence (http://www.Arabidopsis.org, http://www.ncbi.nlm.nih.gov). A *Not*I site at the 5′ end of the forward primer and a *Sbf* I site at the 3′ end of reverse primer were introduced. The remaining steps of plasmid construction were performed as described elsewhere (Ren et al., 2012).

### Quantitative real-time PCR and semi-RT PCR

Samples were collected and frozen in liquid nitrogen for total RNA extraction using the RNAprep Pure Plant Kit (TianGen Biotech). Approximately 1 μg of total RNA was then used for reverse transcription. qRT-PCR was performed using the TransStart Top Green qPCRSuperMix (TransgenBiotech) kit, following the manufacturer's protocol and using a two-step method for PCR reaction. The PCR parameters included 94°C pre-denaturation for 30 s, followed by 40 cycles of the following two steps: 5 s at 94°C and 30 s at 60°C. Plant *actin2* was used as constitutive reference. The primers used in the present study were listed in Supplementary Table 1.

EasyTaq DNA Polymerase (TransGen Biotech) was used for semi-RT PCR, following the manufacturer's protocol. The PCR parameters included 94°C pre-denaturation for 5 min, followed by 24 cycles for the following three steps: 30 s at 94°C, 30 s at 55°C, and 30 s at 72°C. This was then followed by incubation for 10 min at 72°C for final extension.

### Generation of promoter-GUS constructs

The genomic DNA of *Arabidopsis* was extracted from *Arabidopsis* Columbia (Columbia-0) using one step Plant DNA Extraction Reagent (BIOTEKE), following the manufacturer's protocol. Based on our previous *PRPS6B:: GUS* construct (Ren et al., 2012), the *PRPS6B* was replaced by the promoter of *AtFKBP12* (1.1 kb) at the *Asis*I and *Not*I restriction sites, respectively. Then *PAtFKBP12:: GUS* construct was transferred into the destination vector, pEarleyGate303, via LR recombination reactions. The primers of P*AtFKBP12* are listed in the Supplementary Table 1.

### Western blotting

Total protein was extracted using a cell lysis buffer for Western and IP (Biyuntian), which contained 20 mMTris (pH7.5), 150 mMNaCl, 1%Triton X-100, sodium pyrophosphate, β-glycerophosphate, EDTA, Na_3_VO_4_, and leupeptin. Approximately 1 mM of phenylmethylsulphonyl fluoride (PMSF), 1 × Protease Inhibitor Cocktail (Sigma), and 1 × Phosphatase Inhibitor Cocktail 3 (Sigma) were added to the cell lysis buffer for Western and IP prior to the extraction process. The supernatant was collected after centrifugation at 12,000 rpm and quantified by using the RC/DC protein assay kit (BioRad). Approximately 40 μg of total protein was loaded onto a 15% SDS-PAGE gel. After electrophoresis and electroblotting, PVDF membranes (GE) were blocked with milk blocking buffer (Cwbio) for 12 h (overnight) at 4°C and then incubated in blocking buffer containing anti-HA or anti-Myc (dilution: 1:1000; Earthox, E022010-01, E022050-01) for 2 h at room temperature. The PVDF membrane was washed three times (10 min each) with PBST (Cwbio). The secondary antibody [HRP, goat anti-mouse IgG (H+L) Earthox, E030110-01] was diluted 1:10,000 with PBST. Incubation and elution of the secondary antibody were performed as previously described. Proteins were detected using the cECL Western Blot Kit (Cwbio).

### Rapamycin sensitivity test

Our previous study have shown that low concentrations of rapamycin severely inhibit the growth of BP12-2 (Ren et al., 2012). In current work, a rapamycin concentration gradient from 0 to 5 μM was added to 0.5 MS medium, and DMSO was used as control. Four kinds of plant seeds, including the WT, BP12-2, *P35S:: AtFKBP12*, and *P35S:: HsFKBP12*were surface-sterilized and sowed on the medium. After 10 days, differences between various treatments and materials were assessed, and fresh weight and root length were measured. Under liquid growth condition, 3 ml 0.5 MS liquid medium with DMSO or rapamycin was added into 6-well plate. The liquid medium was refreshed every 2 days.

### Fresh weight and root growth measurements

Plants were grown on 0.5 MS medium with different TOR inhibitors. After 10 days growth, all Petri dishes were photographed, and the fresh weight of plants was determined by weighing. Root length measurements were repeated four times per treatment by using the ImageJ software, and measurements for fresh weight and lateral root density were repeated four times. The measurement for lateral root initiation index was as described elsewhere (Dubrovsky et al., 2009).

### β-GUS staining

GUS staining was performed as previously described (Menand et al., 2002) at 6 h for *PAtFKBP12:: GUS*, 30 min for *35S:: GUS*, and 1 h for DR5/BP12-OE11.

## Results

### Overexpression of *ScFKBP12* and *HsFKBP12*, but not *AtFKBP12*, rescues arabidopsis rapamycin sensitivity under aerobic growth condition

**Figure 1:**
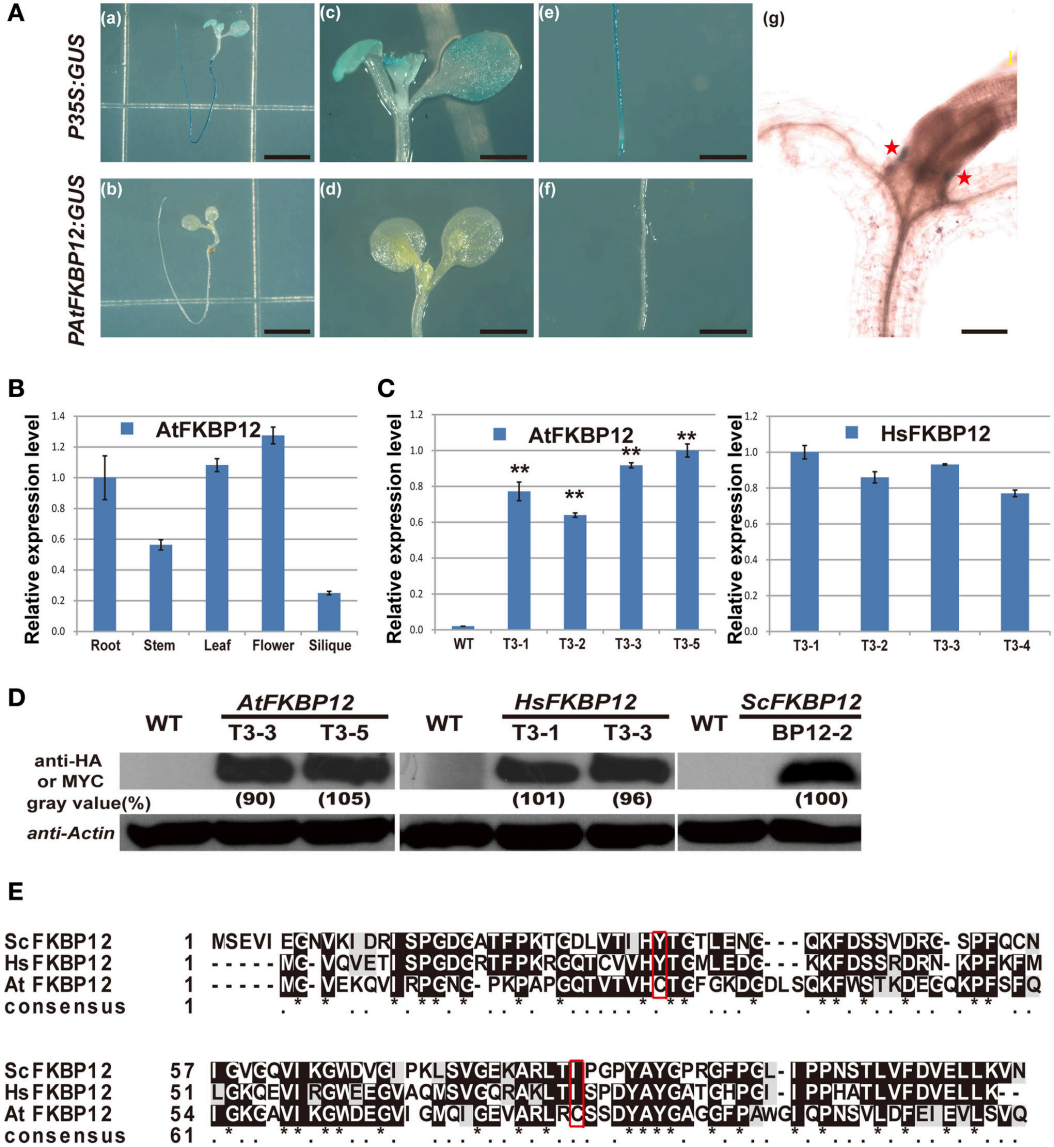
**The expression pattern of *AtFKBP12* in *Arabidopsis* and the identification of transgenic lines of *AtFKBP12*, *HsFKBP12* and *PAtFKBP12::GUS* in *Arabidopsis***. **(A)** The GUS staining of *PAtFKBP12::GUS* and *P35S::GUS* transgenic lines. 5 DAG (days after germination) seedlings were stained. **(a,b)** bar = 0.5 cm; **(c–f)** bar = 2 mm; **(g)** bar = 0.2 mm. **(B)** qRT-PCR to detect expression pattern of *AtFKBP12* in different *Arabidopsis* tissues. **(C)** qRT-PCR to detect expression level of *AtFKBP12* or *HsFKBP12* in transgenic lines. Asterisks denote Student's *t*-test significance compared with WT (^*^P < 0.05;^**^P < 0.01). **(D)** The protein expression level in three *FKBP12* transgenic lines. *AtFKBP12* and *HsFKBP12* used anti-HA antibody; BP12-2 used anti-MYC antibody. **(E)** The multiple protein sequence alignment of ScFKBP12, HsFKBP12, and AtFKBP12. Significant mutations were marked with red box.

Most plants are resistant to rapamycin in solid medium (Xu et al., 1998; Menand et al., 2002; Ren et al., 2012; Montané and Menand, 2013). Two hypotheses are proposed for rapamycin insensitivity in plants. One hypothesis is that rapamycin resistance in plants is due to the low expression of *FKBP12*. Another hypothesis is the currently accepted opinion that plant FKBP12s cannot bind to rapamycin. To study the first hypothesis, the *AtFKBP12* promoter was amplified from *Arabidopsis* genomic DNA, and the *PAtFKBP12::GUS* plasmid was constructed and transformed into WT *Arabidopsis*. A total of 12 independent transgenic plants containing *PAtFKBP12:: GUS* were obtained and confirmed by DNA-PCR using GUS specific primers (Supplementary Table 1). GUS staining showed that the GUS signal of *PAtFKBP12:: GUS* plants was much lower than that of *P35S:: GUS* transgenic plants, which were generated in a previous study (Figure 1A; Ren et al., 2011). Total RNA from different tissues of *Arabidopsis*, including roots, stems, leaves, flowers, and siliques was extracted to determine tissue-specific expression of the *AtFKBP12* gene. qRT-PCR analysis showed that *AtFKBP12*was constitutively expressed in *Arabidopsis* (Figure 1B). The expression level of *AtFKBP12* was higher in the leaves and flowers than in the roots, stems, and siliques.

To further examine whether rapamycin insensitivity in *Arabidopsis* in solid medium results from the low expression of *AtFKBP12*, transgenic lines containing *P35S::AtFKBP12* were prepared. A total of 25 independent lines were identified by PCR analysis of leaf tissue. A total of 14 independent lines (OE1–14) were selected for expression profiling of *AtFKBP12* by semi-qPCR. The *AtFKBP12*transcript was detected in all 14 independent lines, whereas the expression levels of *AtFKBP12*varied among the different lines. Four lines with the highest *AtFKBP12* expression levels were selected for qRT-PCR analysis (Figure 1C). For the rapamycin sensitivity assay, all transgenic lines were grown on solid medium supplemented with 5 μM rapamycin, and the results demonstrated that all lines, including the highest overexpression lines (OE1, OE3, OE5, and OE14) and the lowest overexpression lines (OE2 and OE6) were insensitive to rapamycin under aerobic condition (Table 1). These findings indicate that the expression level of AtFKBP12 was not related to rapamycin insensitivity in plants. This observation was consistent with most previous studies (Xu et al., 1998; Menand et al., 2002; Mahfouz et al., 2006; Sormani et al., 2007; Ren et al., 2012; Montané and Menand, 2013).

**Table 1:** **Screening and identification of transgenic material**.

**Transgenic lines**	**Leaf PCR test positive**	**Rapamycin sensitivity test**	**Rapamycin sensitivity test positive**
*PAtFKBP12::GUS*	20	–	–
*AtFKBP12*-OE	25	14	0
*HsFKBP12*-OE	27	14	14
DR5/BP12-OE	20	6	6

*OE means overexpression*.

**Figure 2:**
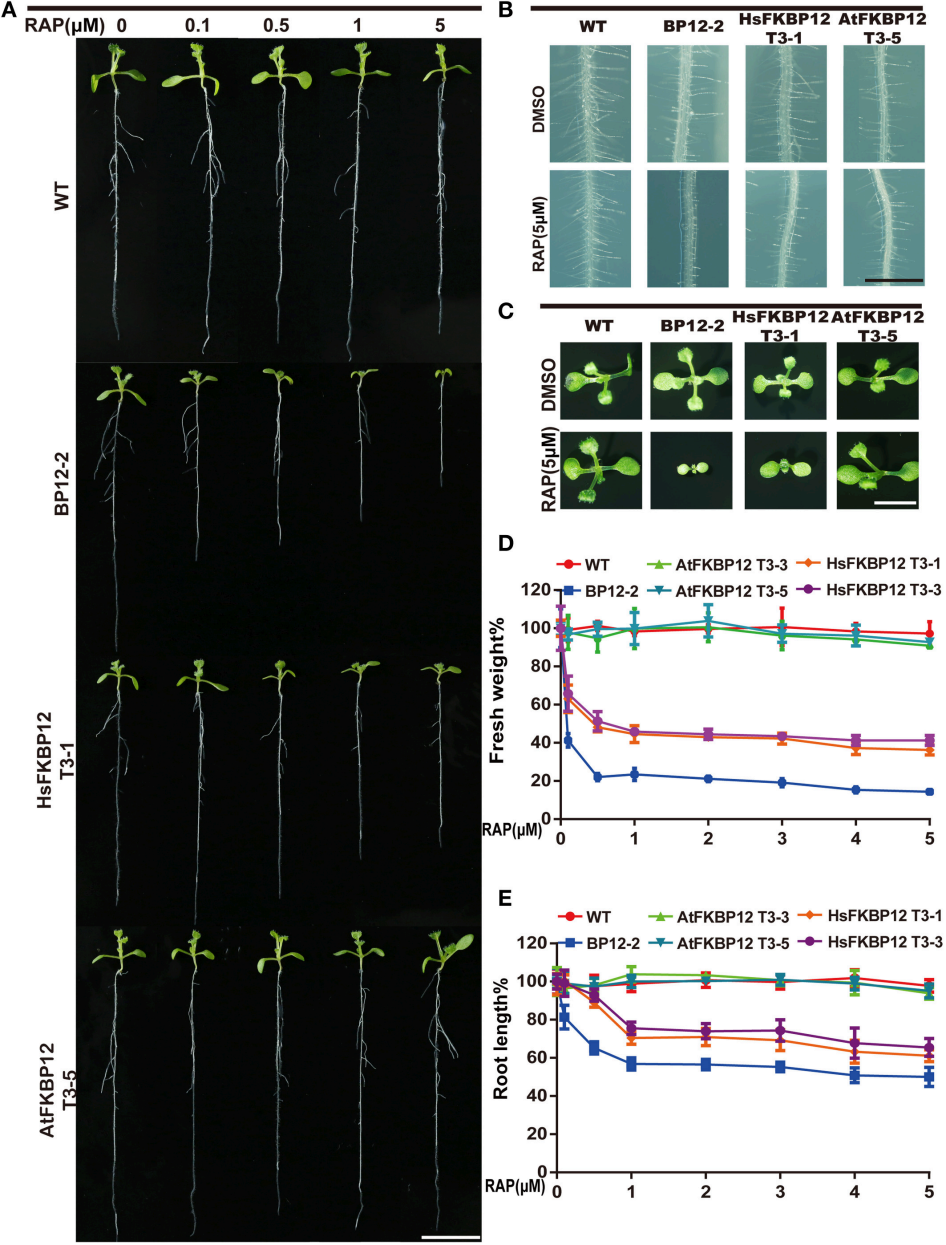
**Rapamycin sensitivity tests of the transgenic plants with *ScFKBP12*, *HsFKBP12*, and *AtFKBP12*. (A)** Dose-dependent effect of rapamycin on the growth and development of WT and three transgenic plants. The concentration of rapamycin ranged from 0 to 5 μM (10 DAG). Bar = 1 cm. **(B,C)** The root hair and leaf growth of WT and three transgenic plants on 0.5 MS medium supplied with DMSO or rapamycin (5 μM). **(B)** Bar = 1 mm; **(C)** Bar = 0.5 cm. **(D,E)** Quantitative analysis and comparison of the root length and the fresh weight (%) of WT and three transgenic plants after treatment with rapamycin. Error bars indicate ±SD for quadruplication.

To verify this result, we cloned *HsFKBP12* from human cells and generated 25 independent *P35S::HsFKBP12* transgenic lines. OE1–OE14 were selected for semi-qPCR and qRT-PCR analyses and treated with 5 μM rapamycin similar to that of AtFKBP12 lines (Table 1, Figure 1C). In contrast to the *P35S::AtFKBP12* lines, all 14 *P35S::HsFKBP12* lines (OE1-OE14) responded to rapamycin (Table 1). In previous studies, we have extensively characterized the rapamycin sensitivity of BP12-2, which has a single copy of *P35S::ScFKBP12* and is hypersensitive to rapamycin (Ren et al., 2012). In the present study, AtFKBP12-OE3, AtFKBP12-OE5, HsFKBP12-OE1, HsFKBP12-OE3 showed higher expression levels among all tested transgenic lines of AtFKBP12 and HsFKBP12, respectively. Western blot results confirmed that the expression of AtFKBP12 and HsFKBP12 proteins were similar and significantly higher (Figure 1D and Supplementary Figure 1A). We therefore selected them for subsequent rapamycin sensitivity testing using procedures described earlier. Figure 2 shows that all tested lines had normally growth on 1/2MS solid medium with DMSO, indicating that overexpression of AtFKBP12, ScFKBP12, and HsFKBP12 had no obvious effect on plant growth. However, in the presence of rapamycin, BP12-2 plants showed hypersensitivity, followed by HsFKBP12-OE1 and OE3, whereas the AtFKBP12 transgenic lines and WT plants did not respond to rapamycin treatment (Figure 2A). The administration of a low concentration of rapamycin (0.1 μM) resulted in a delay in the growth and development of BP12-2, and its fresh weight decreased by nearly 50% compared to the WT (Figure 2E). Administration of 0.1 μM rapamycin did not result in strong detectable inhibitory effects on HsFKBP12-OE1 and OE3. However, with increased rapamycin concentration, the HsFKBP12 transgenic lines also exhibited strong inhibitory effects (Figure 2A). When the concentration of rapamycin reached to 5μM, its inhibitory effect on HsFKBP12-OEs was pronounced (Figures 2A–C). Root and root hair length significantly decreased, leaf size was reduced, and plant fresh weight decreased by >50% (Figures 2D,E). At high concentration of rapamycin, the growth suppression of BP12-2 was still stronger than in HsFKBP12-OE lines. In contrast, the *P35S::AtFKBP12* transgenic lines did not show clear inhibitory effects and had phenotypes similar to those of the WT control (Figure 2).

### Submergence can partially restore rapamycin sensitivity of *AtFKBP12* in *arabidopsis*

Unlike above observations, previous study showed that AtFKBP12 is able to bridge the interaction between rapamycin and TOR in liquid culture (Xiong and Sheen, 2012). To further examine this observation and the functions of ScFKBP12, HsFKBP12 and AtFKBP12 in parallel under anaerobic growth condition, the sterile seeds of WT, BP12-2, HsFKBP12-OE1 and AtFKBP12-OE5 have been germinated in 0.5xMS liquid medium containing 10 μM rapamycin and grown for 8 days, respectively. The Supplementary Figure 2 showed that the seedling growth of BP12-2, HsFKBP12-OE1, and AtFKBP12-OE5 were indistinguishable from WT plants in terms of its response to submergence in 0.5xMS liquid medium with DMSO. However, the cotyledon greening, primary root elongation and fresh weight of BP12-2, followed byHsFKBP12-OE1,have been significantly arrested in the presence of rapamycin (Supplementary Figure 2). These results indicate that the combination of rapamycin and hypoxia could generate additive inhibition effects on TOR activity which are in agreement with recent observations in mammals (Bedogni et al., 2005; Agarwal et al., 2016; Damerill et al., 2016). Importantly, submergence can partially inhibit seedling growth of AtFKBP12-OE5 when compared with WT in the presence of 10 μM rapamycin (Supplementary Figure 2). This observation likely suggests that anaerobic stress caused by submergence can partially rescue the functions of AtFKBP12 to interact with TOR and rapamycin.

### Generation of double transgenic DR5/BP12 plants containing both *P35S*::*ScFKBP12* and DR5:: *GUS*

**Figure 3:**
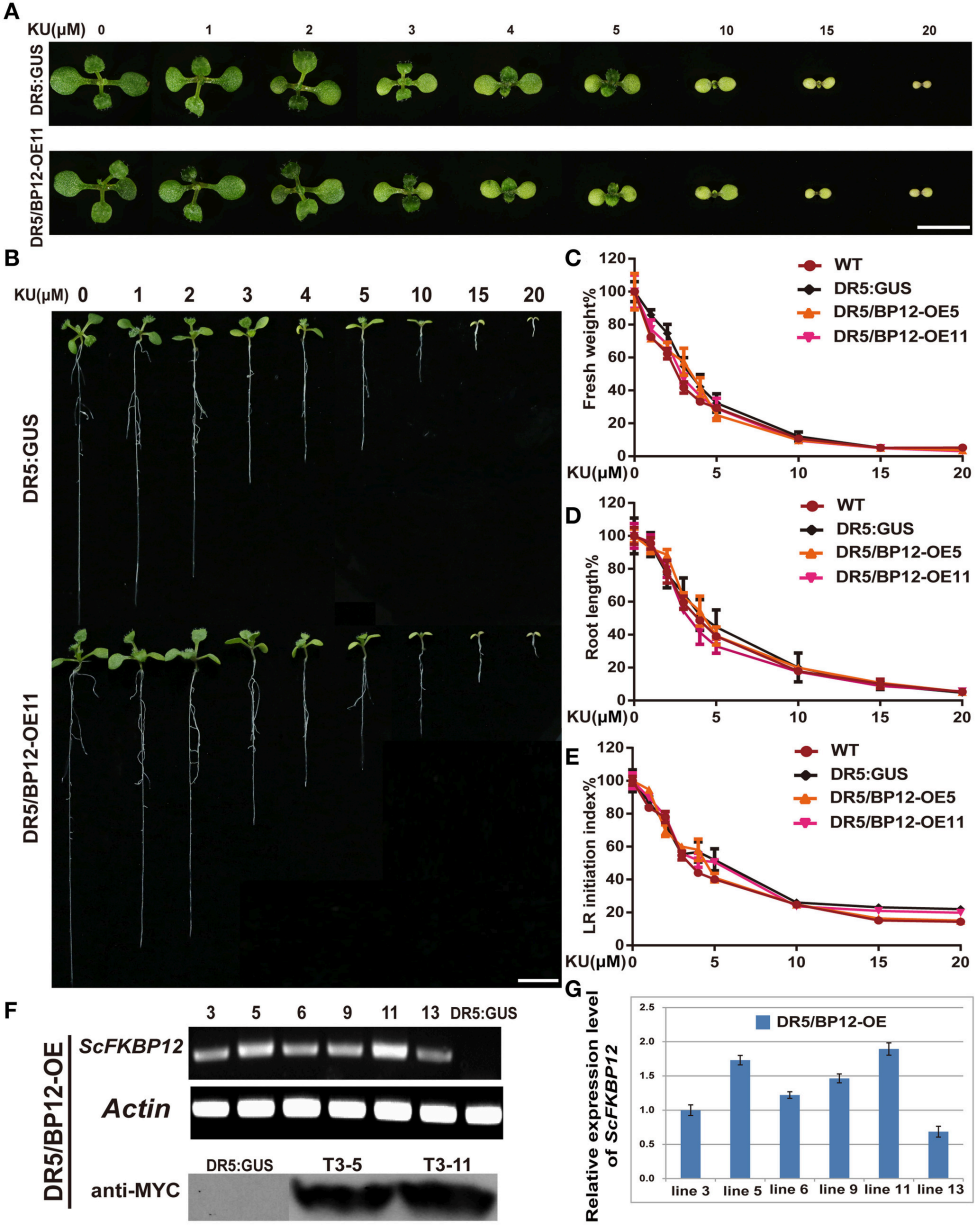
**KU sensitivity tests of WT, *DR5::GUS* and *DR5/BP12-OE* transgenic lines and the identification of transgenic lines of *DR5/BP12-OE* transgenic lines. (A)** The leaf formation of *DR5::GUS* and DR5/BP12-OE11 after treatment with different concentration of KU (10 DAG). Bar = 0.5 cm. **(B)** Dose-dependent inhibitory effect of KU on WT, *DR5:: GUS* and DR5/BP12-OE plants growth. The KU concentration ranged from 0 to 20 μM (10 DAG). Bar = 1 cm. **(C–E)** The quantitative analysis and comparison of fresh weight (%), root length (%) and lateral root initiation index (%) (LR initiation index) of *DR5::GUS* and DR5/BP12-OE plants after treating with KU at different concentration. Error bars indicate ±SD for quadruplication. **(F,G)** Identification of transgenic lines of DR5/BP12-OE lines by semi-qPCR, western blot (**F**, note: Since some results of the western blot located between DR:GUS and T3-5 are not associated with this manuscript, part of the membrane has been cut out), and qRT-PCR **(G)**.

Differing observations on the relationship between TOR and auxin signaling were reported by different groups (Dinkova et al., 2000; Beltrán-Peña et al., 2002; Schepetilnikov et al., 2013; Xiong et al., 2013; Dong et al., 2015). The above results indicated that under aerobic and anaerobic growth conditions, the rapamycin sensitivity of *Arabidopsis*canbe rescued by *ScFKBP12* in the most significant manner. *ScFKBP12* was therefore selected for subsequent analysis of TOR signaling in *Arabidopsis*. To further investigate the relationship between TOR and auxin, we introduced the *P35S:: ScFKBP12* construct (with a BASTA selection marker) into *DR5:: GUS* homozygous *Arabidopsis* lines, which have been used as an auxin signal reporter (Xiang et al., 2011). A total of 20 independent double transgenic lines containing both *DR5:: GUS* and *P35S:: ScFKBP12* were generated by screening T_0_ transgenic seeds sown on 0.5 MS medium plate containing BASTA (10mg/L). DNA-PCR analysis indicated that these lines contained both *DR5::GUS* and *P35S::ScFKBP12* constructs. We named these double transgenic plants as DR5/BP12-OE lines (Table 1). The T2 generation seeds of these 20 DR5/BP12-OE lines were collected for the BASTA segregation assay. The results showed that 6 of 20 lines displayed a 3:1 Mendelian segregation ratio for BASTA resistance and sensitivity, indicating that these 6 lines likely contained a single copy of *P35S:: ScFKBP12*. T3 generation seeds of these 6 lines were then selected for rapamycin sensitivity testing, which showed that all 6 lines were similarly sensitive to rapamycin treatment (Table 1). Semi-qPCR and qRT-PCR analyses indicated that DR5/BP12-OE5 and DR5/BP12-OE11 exhibited higher levels of ScFKBP12 expression than the other lines (Figures 3F,G). They were therefore selected for subsequent experiments.

### The combination of low-concentration rapamycin and KU significantly inhibits seedling growth and development

When DR5/BP12-OE5 and DR5/BP12-OE11 were treated with concentrations of rapamycin ranging from 5 to 20 μM, the plants showed no obvious growth differences related to increasing of rapamycin concentration. So 5 μM seemed to be the saturation concentration of rapamycin (Supplementary Figure 3). This result indicated that rapamycin alone did not inhibit TOR function, even at higher concentrations. To proceed further, studies using second-generation TOR inhibitors to rapamycin-BP12-2 systems should be conducted. AsTORis have been developed and extensively utilized as second-generation TOR inhibitors both in basic scientific research studies and clinical trials (Benjamin et al., 2011). AsTORis directly interact with the kinase domain of TOR by competing with ATP, thereby inhibiting the kinase activity of TOR. More than 30 asTORis have been developed to date. Different asTORis have different structures and different IC50 (half maximal inhibitory concentration). KU63794 (KU), Torin1 and AZD8055 represent mild, moderate and strong inhibitors of TOR, respectively. They have frequently been used in TOR studies on mammalian systems and in plants (Montané and Menand, 2013; Schepetilnikov et al., 2013; Xiong et al., 2013; Dong et al., 2015; Li et al., 2015). However, Torin1 and AZD8055 show some off-target effects on PI3Ks (Thoreen et al., 2009; Chresta et al., 2010; Liu et al., 2010). In contrast, KU displayed the highest specificity toward TOR with no detectable off-target effects (García-Martínez et al., 2009). KU was therefore selected as an ATP competitive inhibitor of TOR for this study.

The inhibitory effect of KU was initially tested using concentration-gradient treatments (Figure 3). KU inhibited plant growth in a dose-dependent manner. When the KU concentration was <2 μM, root length of DR5/BP12-OE5 and DR5/BP12-OE11 was reduced compared to the DMSO control but no obvious differences in leaf development were observed. With higher (10–20 μM) KU concentrations, strong inhibitory effects were observed. Root development in *DR5::GUS*, DR5/BP12-OE5, and DR5/BP12-OE11 was inhibited. Leaf formation was impaired, and the true leaves were smaller and chlorotic (Figures 3A,B). The fresh weight of the whole plant decreased significantly. The 20 μM KU concentration completely inhibited plant growth. Phenotype and related data analysis (Figures 3A–E) indicated that the concentration range of 3–5 μM was critical to plant growth, and possibly the IC50 of KU.

**Figure 4:**
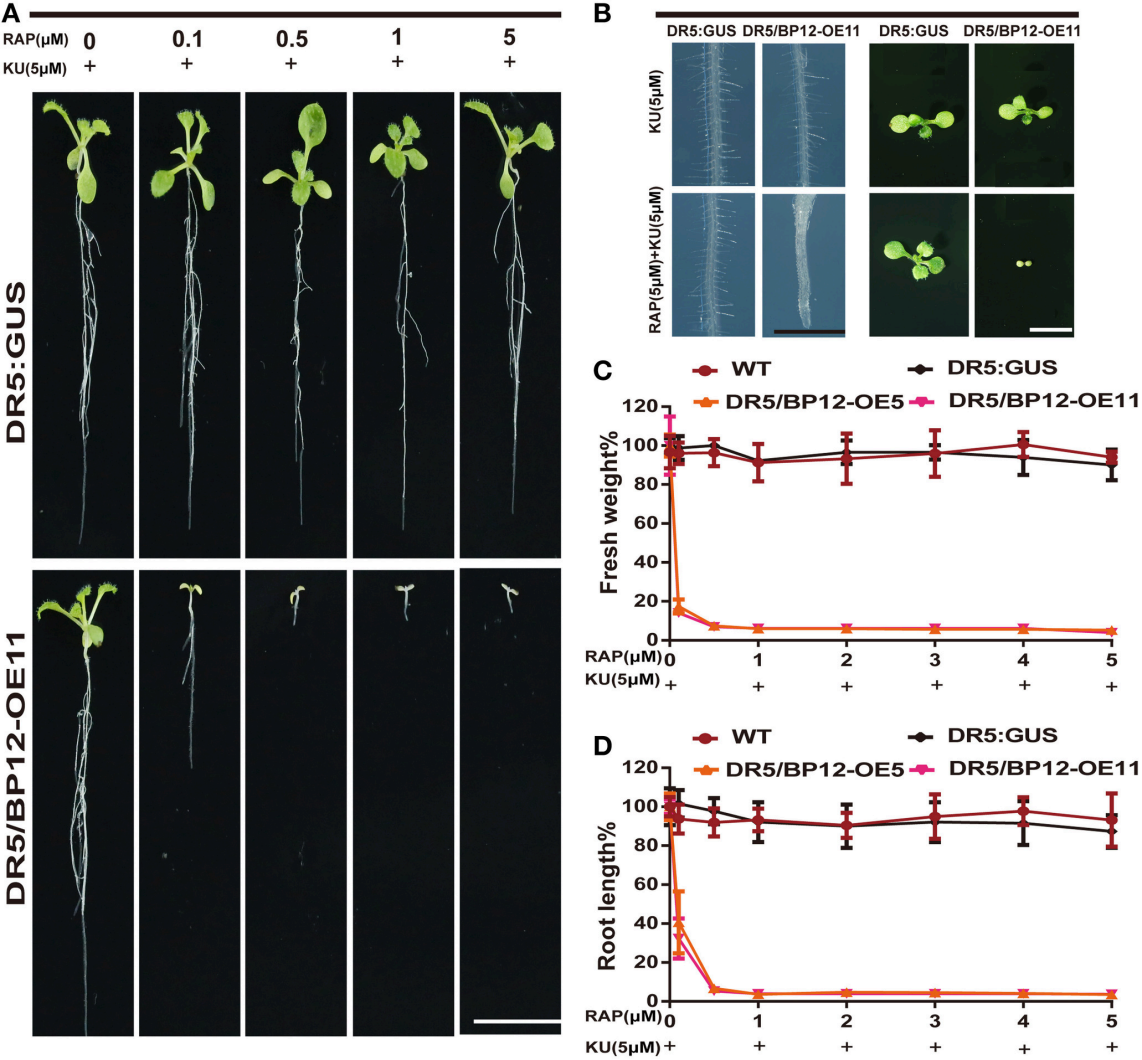
**The inhibition of *Arabidopsis* by combined rapamycin with KU. (A)** The growth of whole plant of *DR5:: GUS* and DR5/BP12-OE11 after treated with rapamycin and KU (10 DAG). Rapamycin concentration ranged from 0 to 5 μM, whereas KU was used at a final concentration of 5 μM. Bar = 1 cm. **(B)** The inhibitory effect of KU or rapamycin plus KU on root hair development and leaf formation of *DR5:: GUS* and DR5/BP12-OE11. Bar = 1 mm at the left and 0.5 cm in the right. **(C,D)** The quantitative analysis and comparison of root length (%) and fresh weight (%) of WT, *DR5::GUS* and DR5/BP12-OE plants after treatment with rapamycin or/and KU. Error bars indicate ±SD for quadruplication.

We then tested various combinations of rapamycin and KU. Rapamycin combined with KU in the medium produced inhibition in the development of DR5/BP12-OE5 and DR5/BP12-OE11 in a dose-dependent manner (Supplementary Figure 4). When the concentration of KU remained at 1 μM, the WT lines showed no inhibitory effect at any rapamycin concentration, whereas DR5/BP12-OE5 and DR5/BP12-OE11showed severe inhibitory effects on root length (Supplementary Figures 4A,D), leaf development (Supplementary Figure 4B), and fresh weight (Supplementary Figure 4D). In contrast, the combination of 5 μM KU with gradually increased rapamycin concentrations resulted in a more pronounced inhibitory effect that was phenotypically expressed in the DR5/BP12-OEtreated lines (Figure 4A). Comparison of the combination of 1 μM KU plus rapamycin with 5 μM KU plus rapamycin showed that the latter combination had greater growth inhibition (Figure 4). With 0.5 μM rapamycin and 5 μM KU, growth and development of DR5/BP12-OE5andDR5/BP12-OE11 was completely terminated, and nearly no root growth or true leaf formation were observed.

### TOR inhibitors affect *Arabidopsis* auxin signals

**Figure 5:**
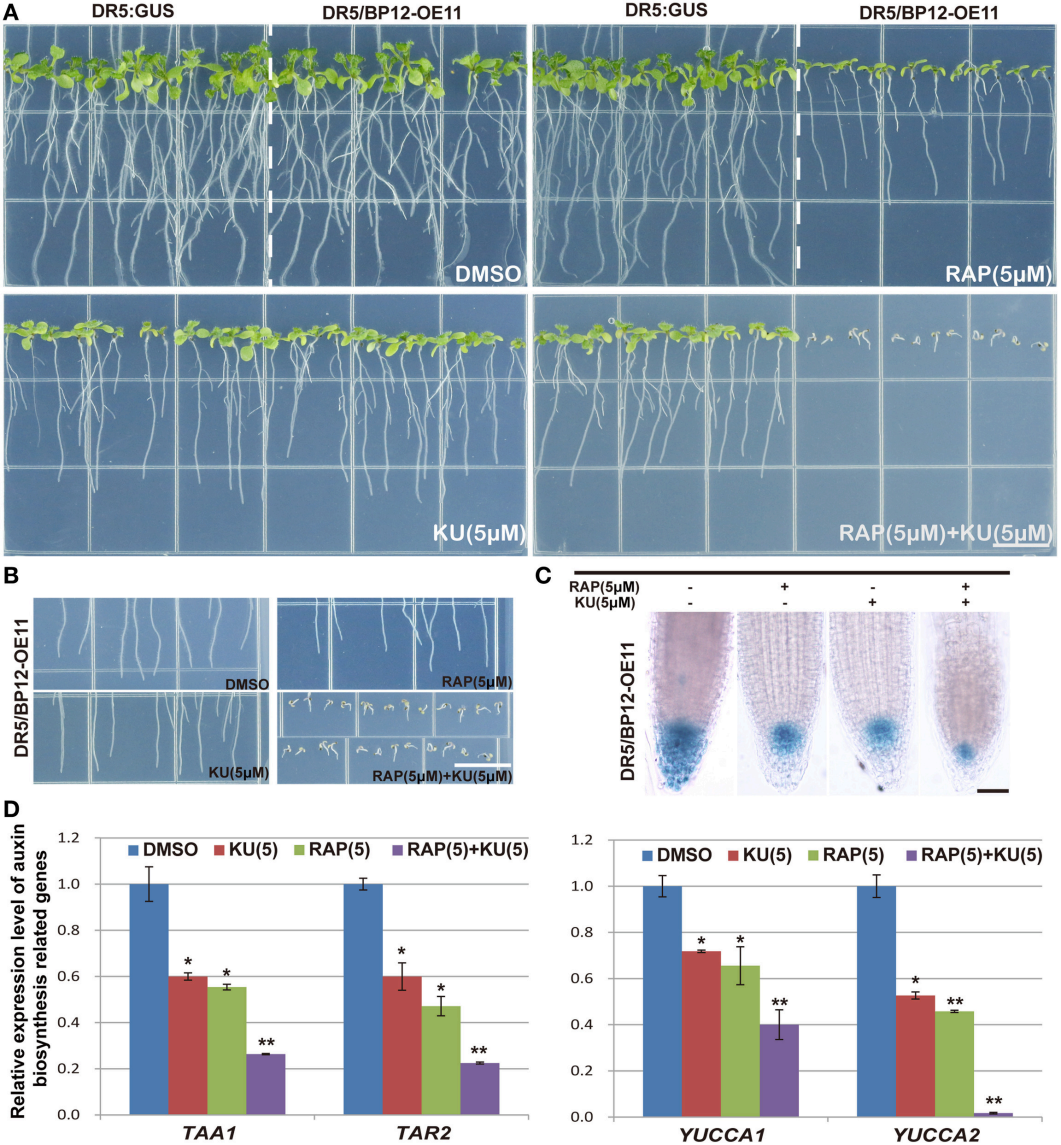
**The inhibition of *DR5:: GUS* and DR5/BP12-OE11 by rapamycin or/and KU affect auxin levels. (A)** The phenotype of *DR5::GUS* and DR5/BP12-OE11 treated with rapamycin or/and KU. DMSO was used as control (12 DAG). Bar = 1 cm. **(B)** The changed gravitropism of plant root. Plants grew for 12 din 0.5 MS medium with different TOR inhibitors or DMSO. Bar = 1 cm. **(C)** Rapamycin or/and KU affect auxin distribution in root tip, *DR5:: GUS* reporter was used as a marker of auxin distribution. Plants grew for 7 d in 0.5MS medium with different TOR inhibitors or DMSO. Bar = 0.1 mm. **(D)** Detection expression level of auxin synthesis-related genes by qRT-PCR. DR5/BP12-OE11 grew 12 days in 0.5 MS medium with different TOR inhibitors [RAP (5 μM), KU (5 μM), RAP (5 μM) +KU (5 μM); DMSO was used as control]. Each value represents the mean± SD of 3 independent experiments. Asterisks denote Student's *t*-test significance compared with control (^*^P < 0.05;^**^P < 0.01).

**Figure 6:**
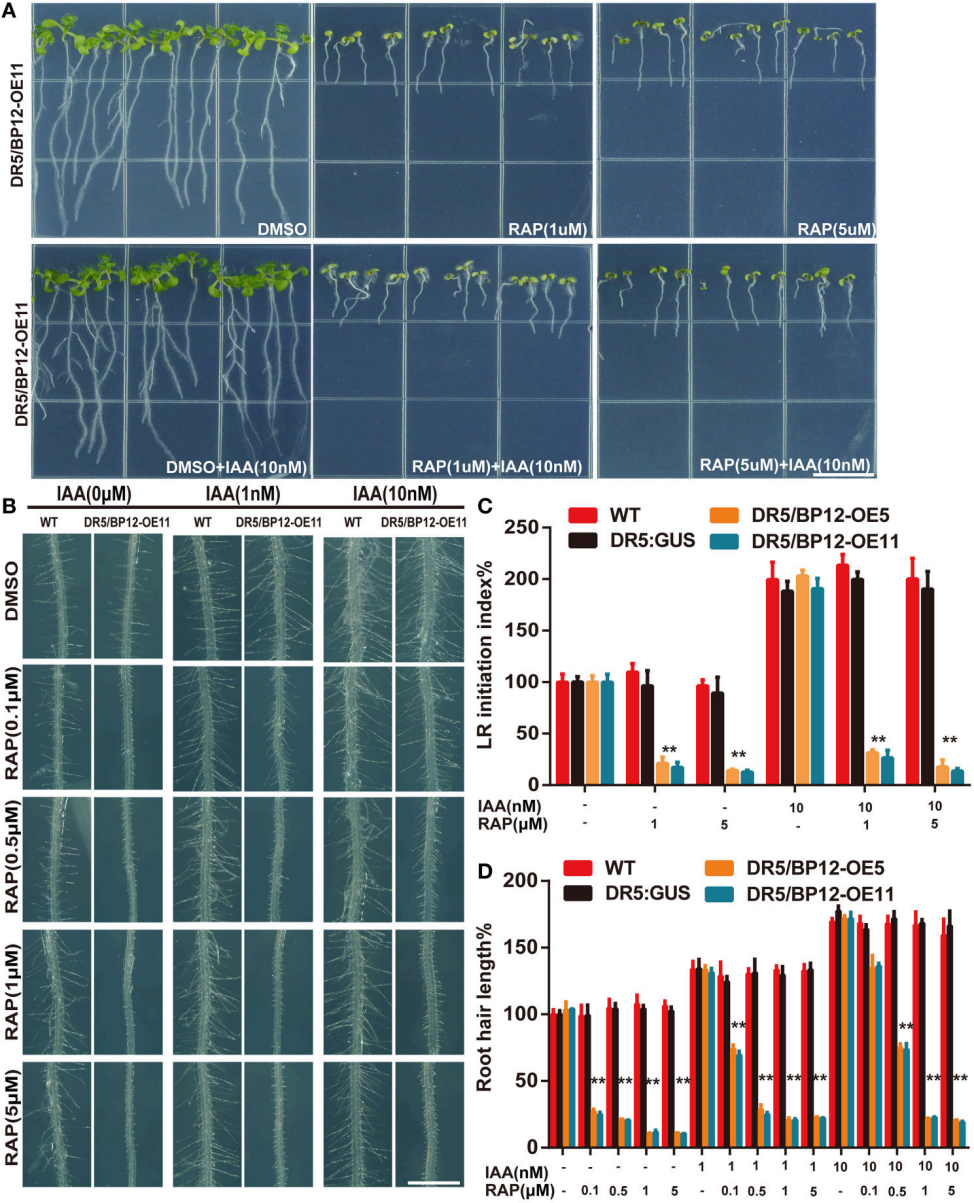
**TOR was required for exogenous IAA response in *Arabidopsis*. (A)** The phenotype of DR5/BP12-OE11 treated with IAA and rapamycin (8 DAG). Bar = 1 cm. **(B)** The root hair elongation of *DR5:: GUS* and DR5/BP12-OE11 with exogenous IAA and rapamycin. Bar = 1 mm. Error bars indicate ±SD for quadruplication. **(C,D)** The quantitative analysis and comparison of LR initiation index (%) and root hair length (%) of WT, *DR5::GUS* and DR5/BP12-OE plants after treatment with IAA and rapamycin. Error bars indicate ±SD for quadruplication. Asterisks denote Student's *t*-test significance compared with *DR5:: GUS* (^**^P < 0.01).

Auxin related plant effects could be distinguished from DR5/BP12-OE11 treated with rapamycin plus KU (Figures 5A,B). Severe growth and development defects, as well as geotropism in DR5/BP12-OE5 and DR5/BP12-OE11 were seen when the combined concentration of rapamycin and KU reached 5 μM. These findings suggested that severe inhibition of TOR would also affect the auxin signal pathway of plants. To investigate inhibitory effects of TOR on auxin, we examined the GUS signals of DR5/BP12-OE11 treated with rapamycin or/and KU (5 μM), with DMSO used as control (Figure 5C). The content of auxin in the root tips of inhibitor-treated lines significantly decreased compared to the control, indicating that the auxin levels were affected by TOR inhibitors. Next, four auxin biosynthesis-related genes, *ATT1, TAR2, YUCCA1*, and *YUCCA2*, were selected for mRNA expression analysis following TOR inhibitor treatment. A*TT1* and *TAR2* are genes involved with the first step of auxin biosynthesis. They catalyze the formation of indole-3-pyruvic acid (IPA) from tryptophan. *YUCCA1* and *YUCCA2* catalyze the formation of IAA from IPA (Mashiguchi et al., 2011). Compared to the DMSO control lines, *ATT1, TAR2, YUCCA1*, and *YUCCA2* were all significantly down regulated in TOR inhibitor-treated lines (Figure 5D and Supplementary Figure 4E). The TOR inhibitors therefore reduced the auxin levels in *Arabidopsis*. The observed down-regulation of auxin levels was also consistent with the disruption in root development in TOR inhibitor-treated DR5/BP12-OE5 and DR5/BP12-OE11. We added exogenous auxin to assess the response of rapamycin-treated DR5/BP12-OE5 and DR5/BP12-OE11. Figure 6 shows that the low concentration of exogenous auxin effectively promoted lateral root and root hair development in the absence of a TOR inhibitor. Under rapamycin treatment, no changes were observed in *DR5:: GUS*, whereas the DR5/BP12-OE5 and DR5/BP12-OE11 lines showed severe impairment of lateral root formation and root hair elongation (Figures 6B–D). The addition of different doses of exogenous auxin resulted in partial restoration of root hair development in low-concentration inhibitor-treated plants (Figures 6B,D). With 5 μM of rapamycin, lateral root development in the DR5/BP12-OE5 and DR5/BP12-OE11 lines was completely inhibited regardless of supplementation with higher amounts of exogenous auxin. These results indicate that TOR is involved in the auxin response.

**Figure 7:**
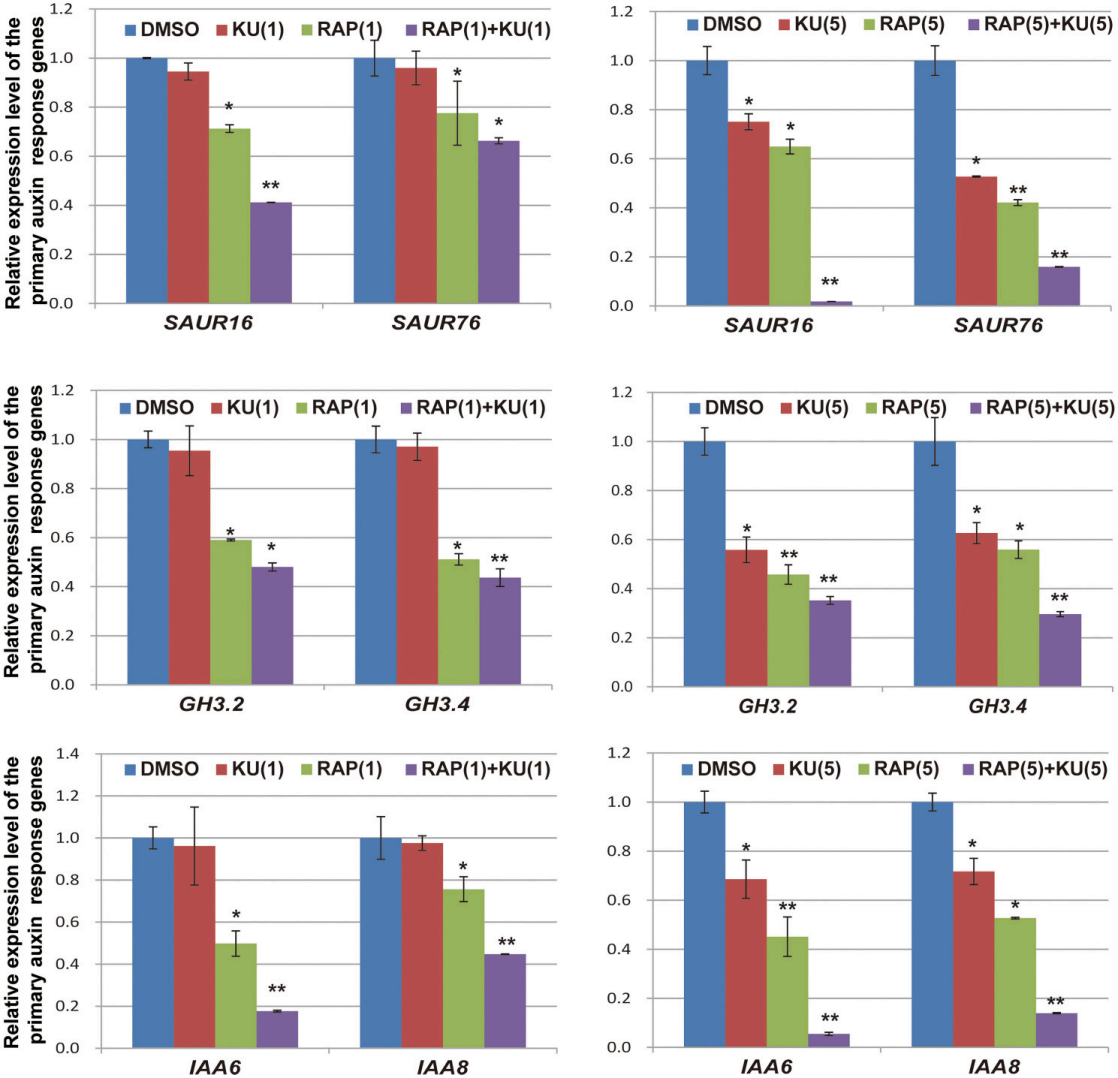
**The expression level of primaryauxin response gene family *AUX/IAAs, SAURs*, and *GH3s* were affected by TOR specific inhibitors**. DR5/BP12-OE11 grew in 0.5 MS medium containing TOR inhibitors [RAP (5 μM), KU (5 μM), RAP (5 μM) + KU (5 μM); DMSO was used as control)] for 12 days. Each value represents the mean± SD of 3 independent experiments. Asterisks denote Student's *t*-test significance compared with control (^*^P < 0.05; ^**^P < 0.01).

The regulatory functions of auxin in plants include apical dominance, tropism, root germination, stem extension, organ differentiation, and aging (Teale et al., 2006; Zhao, 2010). In the presence of auxin, AUX/IAA repressor proteins are rapidly degraded by the ubiquitination pathway and then the transcription of primary auxin response genes (*SAUR, GH3*, and *AUX/IAA* family) are activated within a few minutes (Dharmasiri and Estelle, 2004; Wang and Estelle, 2014). In the present study, we utilized auxin response genes as qRT-PCR markers for auxin responses in DR5/BP12-OE11, which was exposed to TOR inhibitors. Based on previous results of expression profiling analysis (Dong et al., 2015), six genes were selected for gene expression analysis at the mRNA level. Figure 7 shows that all auxin response genes were down-regulated by rapamycin or KU treatment. This differential expression was most obvious when the DR5/BP12-OE11 line was treated with rapamycin plus KU. To further study the effects of TOR inhibitors on auxin signaling, two auxin biosynthesis-related genes and two primary auxin response genes were chosen as markers to study the impact of TOR inhibitors on auxin signals at different time points (Supplementary Figure 5). The results show that the four selected genes were down-regulated at 10 min after treatment. This result indicates that TOR inhibitors could rapidly affect the expression of primary auxin response genes by down-regulation the expression of auxin biosynthesis-related and responsive genes to block auxin signaling. However, *YUCCA* genes and *SAURs* were up-regulated with longer treatment times. This result may be due to the feedback regulation of *YUCCA* genes in the lack of auxin (Suzuki et al., 2015). The up-regulation of *YUCCA* genes might indicate that auxin levels were down-regulated by TOR inhibitors in *Arabidopsis*. In contrast to rapamycin or KU alone, rapamycin plus KU could quickly inhibit auxin signaling and disrupt that feedback system. This might explain why rapamycin plus KU strongly inhibit the growth and development of *Arabidopsis*. The treatment with TOR inhibitors caused a significant change in the transcriptional expression levels of these genes. Therefore, TOR is crucial in auxin homeostasis and significantly affected the auxin signal pathway in plants.

## Discussion

### Hypoxia stress could enhance the interactions between TOR, FKBP12 and rapamycin in *arabidopsis*

Several independent studies have shown that plant FKBP12 is non-functional on solid medium (Xu et al., 1998; Menand et al., 2002; Mahfouz et al., 2006; Sormani et al., 2007; Leiber et al., 2010; Ren et al., 2012), but all these groups did not examine the functions of plant FKBP12s under anaerobic growth condition. Only one group has shown that FKBP12 is functional in during anaerobic growth (Xiong and Sheen, 2012), but they did not set any solid medium control in their experiments. In this study, we re-examined the functions of AtFKBP12 along with HsFKBP12 and ScFKBP12 in *Arabidopsis* under both anaerobic and aerobic growth conditions. Upon submergence, overexpression of FKBP12s can significantly block the growth of *Arabidopsis* in the presence of rapamycin. More importantly, hypoxia caused by submergence can partially rescue the functions of AtFKBP12 to interact with TOR and rapamycin. Since *Arabidopsis* is land plant but not aquatic plant, the biochemical and physiological processes of the submerged seeds and seedlings should be quite different from seeds or seedling growing under aerobic condition. Recent study also revealed that submergence can lead to hypoxia and anaerobic respiration in plant cells (Chen et al., 2015). It is possible that the protein structure of AtFKBP12 can be altered to contribute to the interaction between rapamycin, FKBP12 and TOR under hypoxia stress. However, the underlying mechanism is still remains to be defined. It should be noted that the large amount of literatures already showed that rapamycin treatment can generate the effects similar to hypoxic stress and hypoxia inducible factor-1α (Hif1α), a key mediator of cellular adaptation to hypoxia, can be efficiently inhibited by rapamycin in mammals (Bedogni et al., 2005; Agarwal et al., 2016; Damerill et al., 2016). Based on these observations, it is not surprising that TOR signaling might also act as a key target of hypoxia in *Arabidopsis* as observed in mammals (Hudson et al., 2002; Arsham et al., 2003). Altogether, the combination of rapamycin and hypoxia can generate additive inhibition effects on plant growth in the presence of exogenous or endogenous FKBP12s.

Our results also demonstrated that the rapamycin treatment, *ScFKBP12* transgenic lines showed the strongest drug sensitivity. This might be explained in terms of amino acid sequences (Figure 1E). Amino acid sequence alignment showed relatively high similarity among ScFKBP12, HsFKBP12, and AtFKBP12. The glycine residues and the drug-binding pocket are all conserved (Clardy, 1995). However, two amino acids of AtFKBP12, tyrosine at position 25 and isoleucine at position 79, were changed into cysteine (labeled with a red box in Figure 1E), which affected the protein structure and its biological activity (Xu et al., 1998). The existence of two cysteines might also lead to the formation of a disulfide bond, which would be a critical modification of protein structure and corresponding enzymatic activity. Comparison the amino acid sequence between ScFKBP12 andHsFKBP12, twokey residuesGln^54^and Glu^55^in HsFKBP12interact to rapamycin were changed into Gly^60^ and Gln^61^ in ScFKBP12 (Supplementary Figure 1B; Choi et al., 1996; Sormani et al., 2007). Additionally, many different amino acids were also observed in N-terminal of the proteins. These amino acid differences likely lead to the different affinity rapamycin binding between HsFKBP12 and ScFKBP12 in *Arabidopsis*, but the detailed molecular mechanism remains to be further defined.

### The combination of rapamycin and KU significantly inhibits *arabidopsis* growth

Rapamycin can inhibit TOR activity through rapamycin-FKBP12-FRB/TOR complex formation and display a plateau effect (Sormani et al., 2007; Ren et al., 2012). The second generation TOR inhibitors (asTORis) inhibit TOR by targeting the catalytic site of the kinase domain and competing with ATP in a dose-dependent manner. KU is able to inhibit TORC1 and TORC2 with an IC50 of 10 nM *in vitro* but it inhibits cell growth at 3 μM in MEF cells (García-Martínez et al., 2009) and the IC50 of rapamycin is 0.1nM in HEK293 cells (Edwards and Wandless, 2007). The IC50 values of KU and rapamycin in *Arabidopsis*are ~5 and ~1 μM, respectively. This suggests that the influx/efflux transporters and pharmacological dynamics of TOR inhibitor drugs are quite different in animal and plant cells. Rapamycin and KU precipitates occurred in MS medium solution and plates when the drugs were used at high concentrations (>5 μM). The solubility of KU and rapamycin is poor even in DMSO. This would act to reduce experimental accuracy. An important strategy would be to combine these two different kinds of TOR inhibitors with different inhibitory mechanisms to suppress TOR function. The applications and studies of Torin1, AZD8055, and KU in plants have shown that KU is the most moderate inhibitor, but one with very high TOR specificity. Therefore, we tried the combination of rapamycin with KU to evaluate efficiency of TOR inhibition. We found that TOR is strongly inhibited by a combination of rapamycin and KU at low concentrations (1 μM + 1 μM). A single inhibitor could not produce this level of inhibitory effect at a 10-folds higher concentration. The inhibitor-combination application effectively lowered the dosage of inhibitor and reduced the experiment costs. Plant growth was completely terminated once the concentration of rapamycin and KU was 5 μM. However, the relationship between rapamycin and KU during the actual process of inhibition remains elusive and requires further investigation.

### TOR was required for plant growth in respond to auxin

Auxin plays an important role in the growth and development of plants. Auxin activates TOR and promotes the translation reinitiation of mRNA (Schepetilnikov et al., 2013). The TOR inhibitor Torin1 can interfere with the auxin redistribution (DR5-GFP) in root tips and root gravitropic responses indicating a close connection between TOR and auxin (Dinkova et al., 2000; Schepetilnikov et al., 2013). Some key genes associated with auxin signaling transduction are significantly mis-expressed when TOR is suppressed by AZD8055 (Dong et al., 2015). Although the approaches, TOR inhibitors (rapamycin/KU vs. Torin1) and plant materials (AtFKBP12, HsFKBP12 and ScFKBP12 transgenic plants vs. TOR RNAi plants) we used are quite different between ours and (Schepetilnikov et al., 2013), two independent studies really reach to similar conclusion in which TOR plays a crucial role in the auxin signaling transduction (Schepetilnikov et al., 2013). However, Xiong et al. (2013) demonstrated that auxin is decoupled from TOR signaling activation, and thus auxin signaling (DR5-GFP) doesn't alter when TOR is suppressed (Xiong et al., 2013). These conflicting observations likely caused by different experimental conditions. Xiong et al. (2013), transiently treated the seedlings only for few hours, whereas we and (Schepetilnikov et al., 2013), grow seedlings for several days (Schepetilnikov et al., 2013; Xiong et al., 2013). We observed that the strong growth and gravity defects occurred in root when DR5/BP12-OE5 and DR5/BP12-OE11 was treated with rapamycin plus KU. The auxin distribution significantly decreased in root tip when TOR was suppressed. These results were highly consistent with the observation of Schepetilnikov et al., but not with Xiong et al. (Schepetilnikov et al., 2013; Xiong et al., 2013). Although Xiong et al., emphasized that TOR was decoupled from auxin signaling in Arabidopsis, many auxin signaling associated genes were also differentially expressed in tor seedlings (Xiong et al., 2013). Importantly, some overlapping differentially expressed genes were found in TOR suppression plants by independent groups (Ren et al., 2012; Xiong et al., 2013; Dong et al., 2015), indicating that TOR acts as a key player in auxin signaling transduction in Arabidopsis. However, the underlying molecular mechanism involving TOR and auxin signals remains unclear. With establishment of the DR5/BP12 system in this study, a highly potent, inducible, and selective TOR suppression system is now available for advanced studies on the relationship between TOR and auxin in plants.

## Author contributions

MR, KD, and LY designed the experiments. KD, XZ, KZ, and WW performed the experiments. MR, PD, and JZ analyzed the data. MR, KD, and LY wrote the manuscript text.

## Funding

National Basic Research Program of China (No. 2013CB127100); Fundamental Research Funds for the Central Universities (No. CDJZR14295501); Chongqing Frontier and Applied Basic Research (No. CSTC2014JCYJA80012); the Open Project Funding of State Key Laboratory of Cotton Biology (No. CB2014A08 and CB2015A14); Promote Scientific Research and Cooperation and High level Personnel Training Project in America and Oceania supported by Ministry of Education (No. 0903005109094/003) and National Natural Science Foundation of China (No. 31200903).

### Conflict of interest statement

The authors declare that the research was conducted in the absence of any commercial or financial relationships that could be construed as a potential conflict of interest.

## Supplementary material

Supplementary Figure 1 **The protein expression level in three *FKBP12* transgenic lines (A) and the multiple protein sequence alignment of ScFKBP12, HsFKBP12, and AtFKBP12 (B)**. Key residues were marked with red box.

Supplementary Figure 2 **Rapamycin sensitivity tests of the transgenic plants with *ScFKBP12, HsFKBP12*, and *AtFKBP12* inanaerobic growth conditions (8 DAG). (A)** The effect of rapamycin on the growth and development of WT and three transgenic plants during anaerobic growth condition (8 DAG). Bar = 0.5 cm. **(B)** The leaf growth of WT and three transgenic plants on 0.5 MS liquid medium supplied with DMSO or rapamycin (5 μM). **(B)** Bar = 0.5 cm. **(C)** Quantitative analysis and comparison of the fresh weight (%) of WT and three transgenic plants after treatment with rapamycin. Error bars indicate ±SD for quadruplication. Asterisks denote Student's *t*-test significance compared with control (^*^*P* < 0.05;^**^*P* < 0.01).

Supplementary Figure 3 **Rapamycin cannot completely inhibit DR5/BP12-OE lines growth even with very high concentration (20 **μ**M) (10 DAG)**.

Supplementary Figure 4 **The inhibition of *Arabidopsis* by combined rapamycin with KU. (A)** The growth of whole plant of *DR5:: GUS* and DR5/BP12-OE11 after treated with rapamycin and KU (10 DAG). Rapamycin concentration ranged from 0 to 5 μM, whereas KU was used in a final concentration of 1 μM. Bar = 1 cm. **(B)** The inhibition effect of rapamycin or KU or rapamycin plus KU on root hair development and leaf formation of *DR5::GUS* and DR5/BP12-OE11. Bar = 1 mm at the left and 0.5 cm in the right. **(C,D)** The quantitative analysis and comparison of root length and fresh weight (%) of *DR5::GUS* and DR5/BP12-OE plants after treatment with rapamycin or/and KU. **(E)** Detection expression level of auxin synthesis-related genes by qRT-PCR. DR5/BP12-OE11 grew 12 days in 0.5 MS medium with different TOR inhibitors [RAP (1 μM), KU (1 μM), RAP (1 μM) +KU (1 μM); DMSO was used as control]. Each value represents the mean± SD of 3 independent experiments. Asterisks denote Student's *t*-test significance compared with control (^*^*P* < 0.05;^**^*P* < 0.01).

Supplementary Figure 5 **The expression level of auxin biosynthesis-related genes and primaryauxin response genes were affected by TOR specific inhibitors in short time treatment**. DR5/BP12-OE11grew in 0.5 MS medium for 10 days. Seedlings were transferred into 0.5 MS medium containing TOR inhibitors [RAP (5 μM), KU (5 μM), RAP (5 μM)+KU (5 μM); DMSO was used as control]for different time points (10 min, 30 min, 1 h, 2 h, 3 h, 6 h,12 h, 24 h), then root was collected for RNA extraction. Each value represents the mean ± SD of 3 independent experiments.

Supplementary Table 1 **Primers were used in this study**.
