# Supplementary figures and images for: Target of Rapamycin Is a Key Player for Auxin Signaling Transduction in Arabidopsis
Source: Front Plant Sci. 2016 Mar 11;7:291. doi: 10.3389/fpls.2016.00291 (PMC4786968; doi:10.3389/fpls.2016.00291)

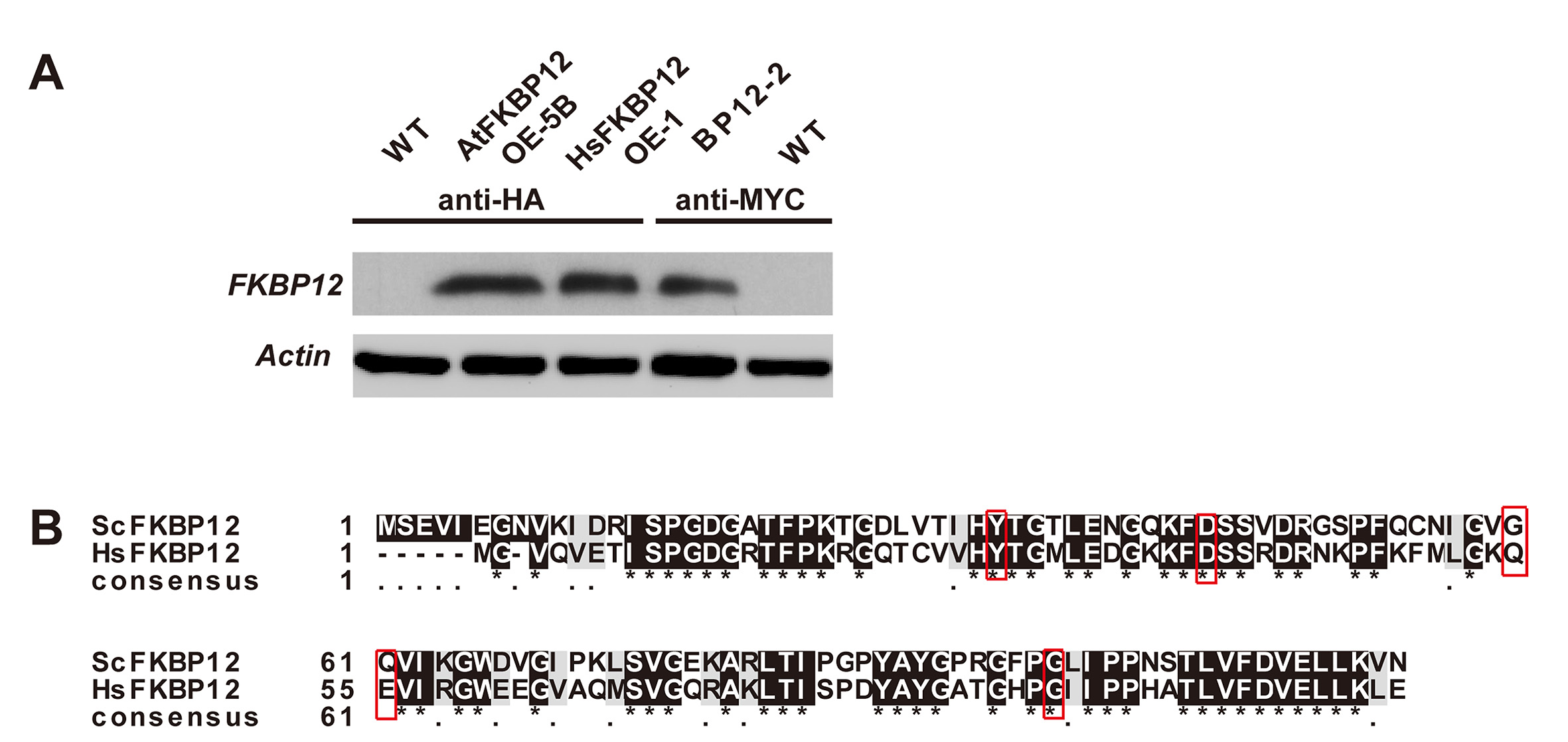

Supplement: Supplementary Figure 1 — The protein expression level in three FKBP12 transgenic lines (A) and the multiple protein sequence alignment of ScFKBP12, HsFKBP12, and AtFKBP12 (B). Key residues were marked with red box. [file Image1.JPEG]

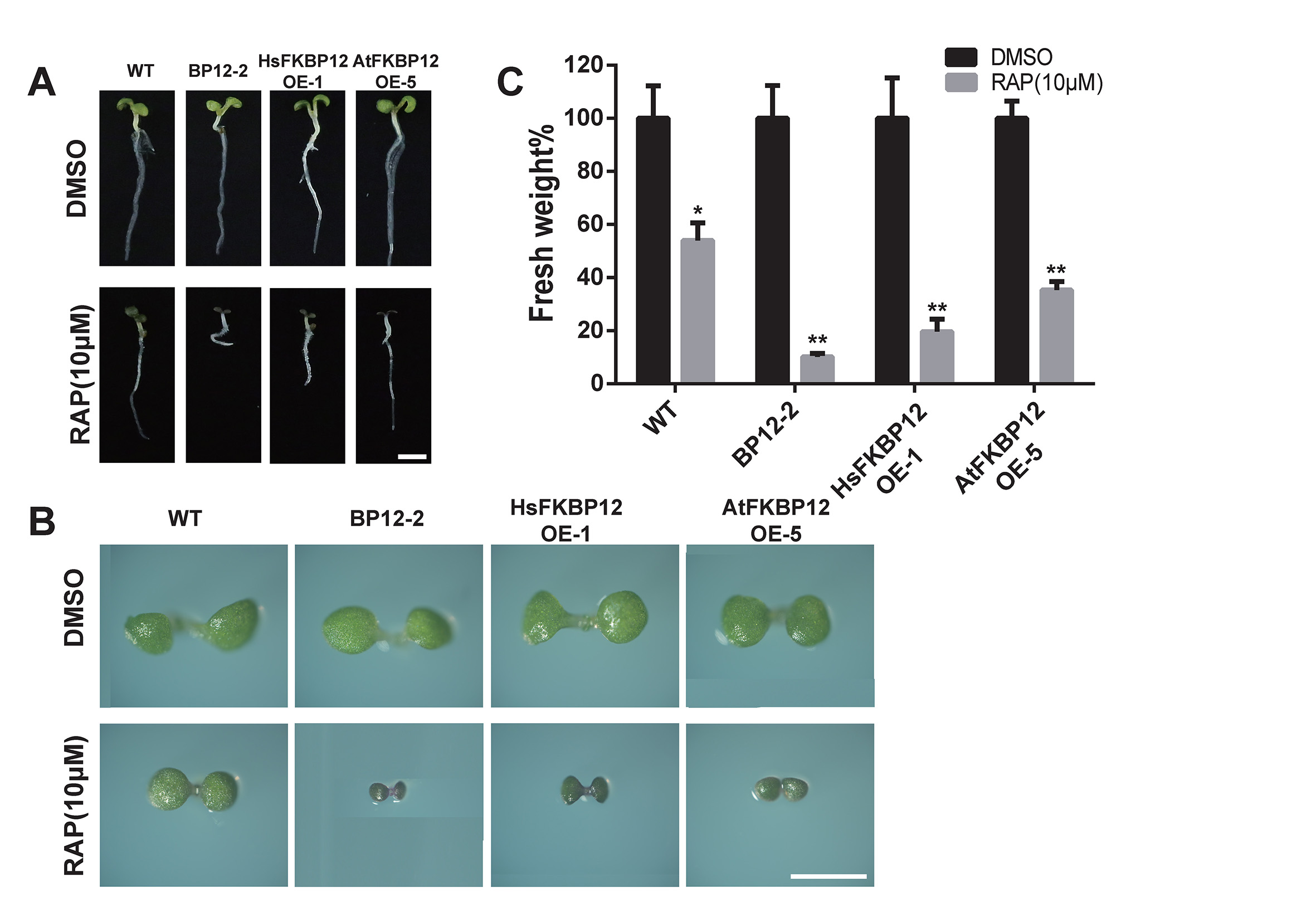

Supplement: Supplementary Figure 2 — Rapamycin sensitivity tests of the transgenic plants with ScFKBP12, HsFKBP12, and AtFKBP12 inanaerobic growth conditions (8 DAG). (A) The effect of rapamycin on the growth and development of WT and three transgenic plants during anaerobic growth condition (8 DAG). Bar = 0.5 cm. (B) The leaf growth of WT and three transgenic plants on 0.5 MS liquid medium supplied with DMSO or rapamycin (5 μM). (B) Bar = 0.5 cm. (C) Quantitative analysis and comparison of the fresh weight (%) of WT and three transgenic plants after treatment with rapamycin. Error bars indicate ±SD for quadruplication. Asterisks denote Student's t-test significance compared with control (*P < 0.05;**P < 0.01). [file Image2.JPEG]

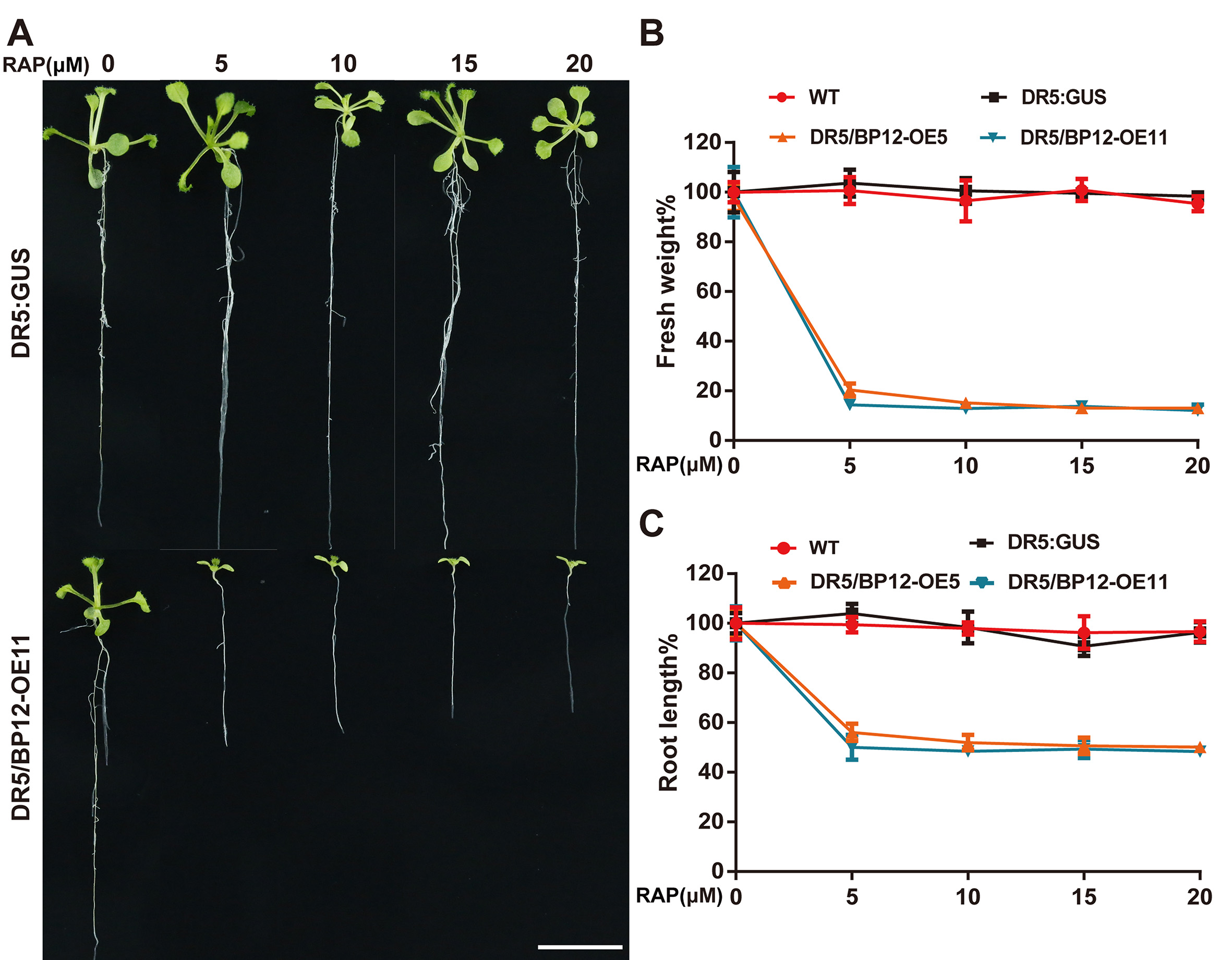

Supplement: Supplementary Figure 3 — Rapamycin cannot completely inhibit DR5/BP12-OE lines growth even with very high concentration (20 μM) (10 DAG). [file Image3.JPEG]

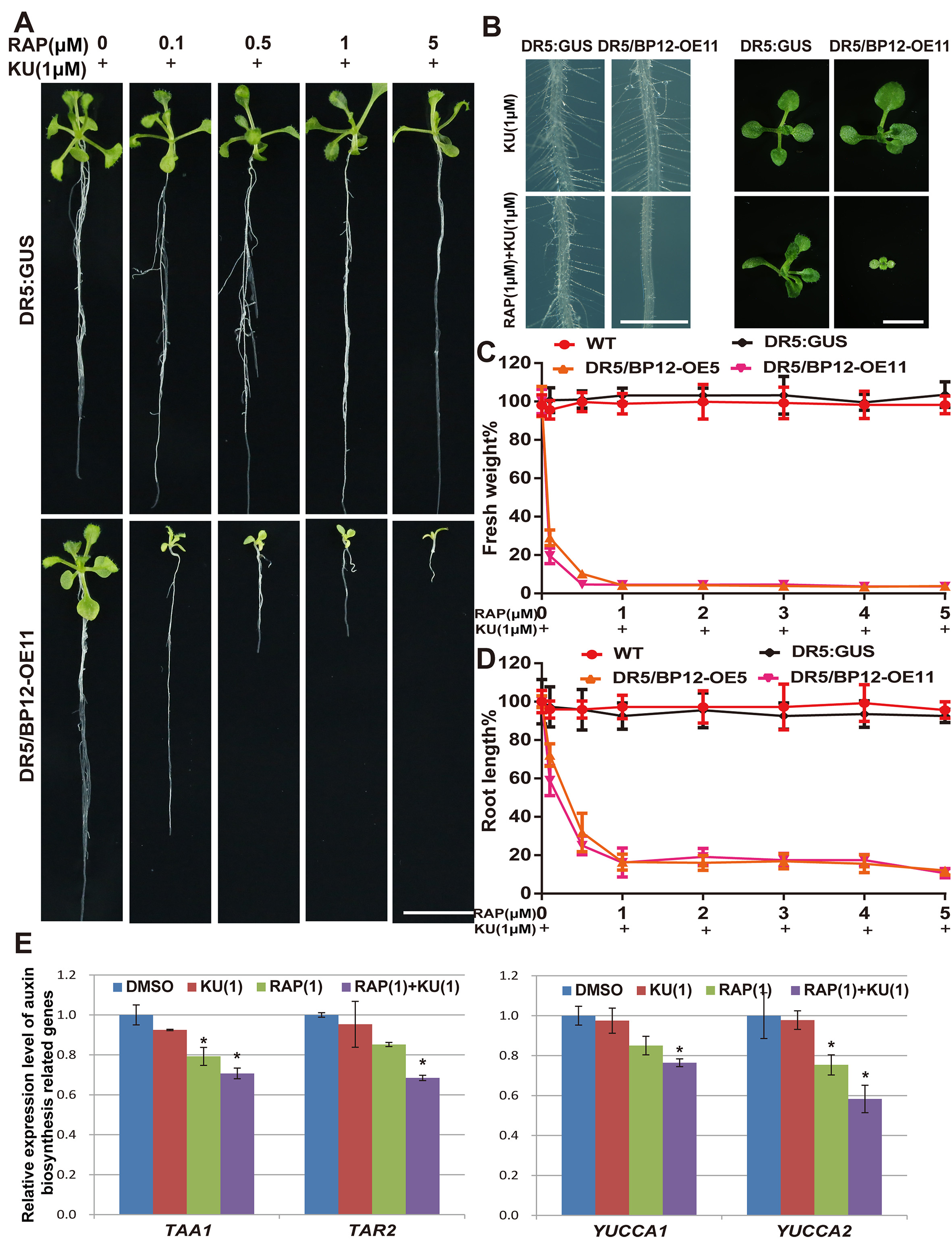

Supplement: Supplementary Figure 4 — The inhibition of Arabidopsis by combined rapamycin with KU. (A) The growth of whole plant of DR5:: GUS and DR5/BP12-OE11 after treated with rapamycin and KU (10 DAG). Rapamycin concentration ranged from 0 to 5 μM, whereas KU was used in a final concentration of 1 μM. Bar = 1 cm. (B) The inhibition effect of rapamycin or KU or rapamycin plus KU on root hair development and leaf formation of DR5::GUS and DR5/BP12-OE11. Bar = 1 mm at the left and 0.5 cm in the right. (C,D) The quantitative analysis and comparison of root length and fresh weight (%) of DR5::GUS and DR5/BP12-OE plants after treatment with rapamycin or/and KU. (E) Detection expression level of auxin synthesis-related genes by qRT-PCR. DR5/BP12-OE11 grew 12 days in 0.5 MS medium with different TOR inhibitors [RAP (1 μM), KU (1 μM), RAP (1 μM) +KU (1 μM); DMSO was used as control]. Each value represents the mean± SD of 3 independent experiments. Asterisks denote Student's t-test significance compared with control (*P < 0.05;**P < 0.01). [file Image4.JPEG]

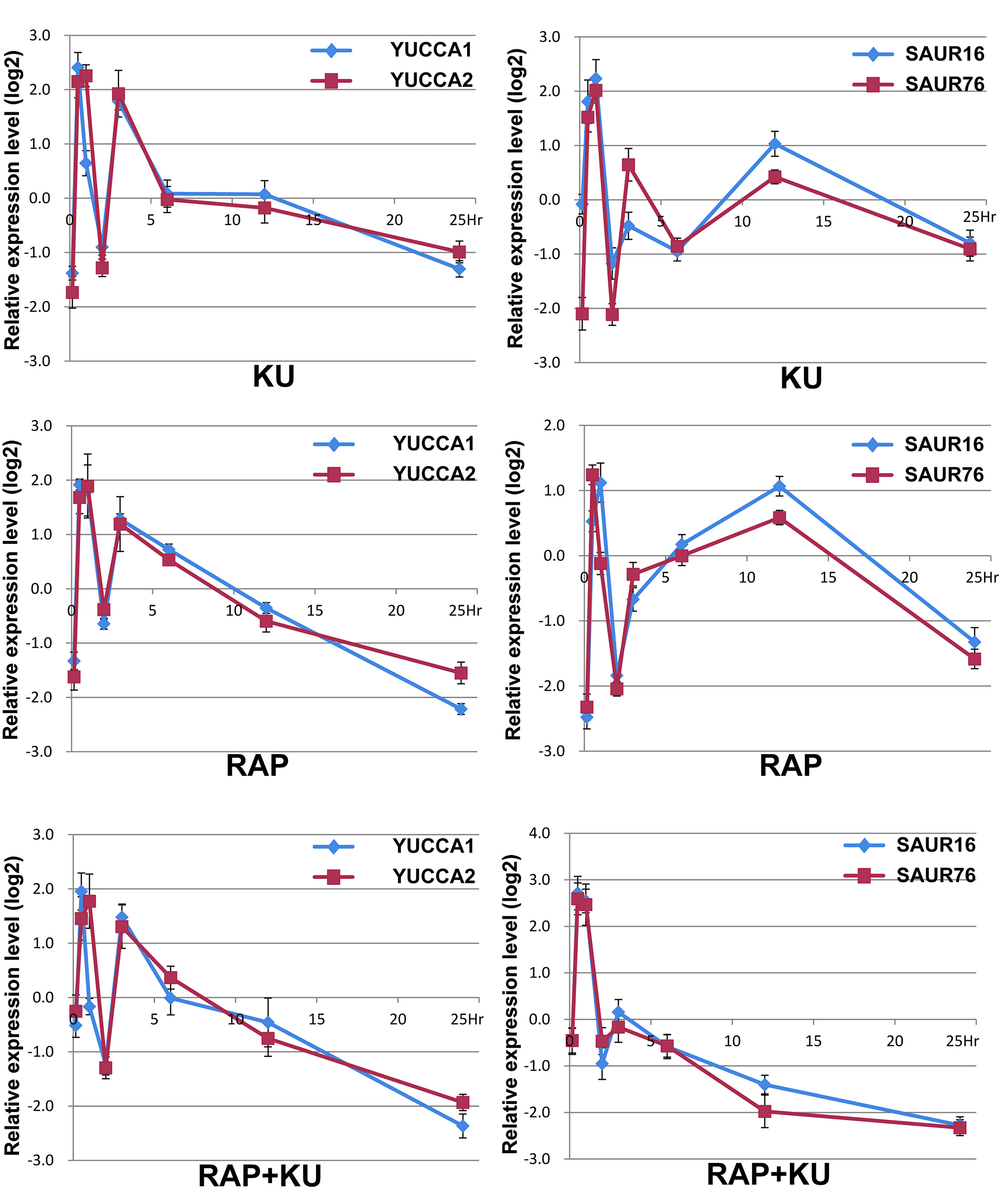

Supplement: Supplementary Figure 5 — The expression level of auxin biosynthesis-related genes and primaryauxin response genes were affected by TOR specific inhibitors in short time treatment. DR5/BP12-OE11grew in 0.5 MS medium for 10 days. Seedlings were transferred into 0.5 MS medium containing TOR inhibitors [RAP (5 μM), KU (5 μM), RAP (5 μM)+KU (5 μM); DMSO was used as control]for different time points (10 min, 30 min, 1 h, 2 h, 3 h, 6 h,12 h, 24 h), then root was collected for RNA extraction. Each value represents the mean ± SD of 3 independent experiments. [file Image5.JPEG]
