# Supplementary material for: Target of Rapamycin Is a Key Player for Auxin Signaling Transduction in Arabidopsis
Source: Front Plant Sci. 2016 Mar 11;7:291. doi: 10.3389/fpls.2016.00291 (PMC4786968; doi:10.3389/fpls.2016.00291)
Supplement: Supplementary Table 1 — Primers were used in this study. [file Table1.docx]

**Supplementary Table 1 ǀ** Primers were used in this study.

| **Cloning primer** |  | |
| --- | --- | --- |
| AtFKBP12F | GCGGCCGCATGGGTGTGGAGAAGCAAGTCATC | |
| AtFKBP12R | CCTGCAGGCTGCACGCTCAGTACTTCGATTTC | |
| HsFKBP12F | GCGGCCGCATGGGAGTGCAGGTGGAAACC | |
| HsFKBP12R | CCTGCAGGTTCCAGTTTTAGAAGCTCCAC | |
| PAtFKBP12F | GCGATCGCGGAATCCCTGTAGTTGGAATCTG | |
| PAtFKBP12R | GCGGCCGCCTCTATATCTTCTCTCTGTCTCTC | |
|  |  | |
| **Leaf PCR primer** |  | |
| 35SF1 | ATGACGCACAATCCCACTATCCTTC | |
| AtFKBP12R | CCTGCAGGCTGCACGCTCAGTACTTCGATTTC | |
| HsFKBP12R | CCTGCAGGTTCCAGTTTTAGAAGCTCCAC | |
| ScFKBP12R | CCTGCAGGGTTGACCTTCAACAATTCGACG | |
| PAtFKBP12F | GCGATCGCGGAATCCCTGTAGTTGGAATCTG | |
| RLGUSR | ACTTGCAAAGTCCCGCTAGT | |
| RLGUSF | CGCAGCGTAATGCTCTACACCAC | |
| RLGUSR | ACTTGCAAAGTCCCGCTAGT | |
|  |  | |
| **Semi-qPCR primer** | |  |
| AtFKBP12F | GCGGCCGCATGGGTGTGGAGAAGCAAGTCATC | |
| AtFKBP12R | CCTGCAGGCTGCACGCTCAGTACTTCGATTTC | |
| HsFKBP12F | GCGGCCGCATGGGAGTGCAGGTGGAAACC | |
| HsFKBP12R | CCTGCAGGTTCCAGTTTTAGAAGCTCCAC | |
| ScFKBP12F | GCGGCCGCATGTCTGAAGTAATTGAAGGT | |
| ScFKBP12R | CCTGCAGGGTTGACCTTCAACAATTCGACG | |
|  |  | |
| **qRT-PCR primer** |  | |
| ScFKBP12F | GCGGCCGCATGTCTGAAGTAATTGAAGGT | |
| ScFKBP12R | CCTGCAGGGTTGACCTTCAACAATTCGACG | |
| AtFKBP12F | GCGGCCGCATGGGTGTGGAGAAGCAAGTCATC | |
| AtFKBP12R | CCTGCAGGCTGCACGCTCAGTACTTCGATTTC | |
| HsFKBP12F | GCGGCCGCATGGGAGTGCAGGTGGAAACC | |
| HsFKBP12R | CCTGCAGGTTCCAGTTTTAGAAGCTCCAC | |
| RLACTIN2F | GCACTTGCACCAAGCAGCAT | |
| RLACTIN2R | CCTTTCAGGTGGTGCAACGAC | |
| RLTAA1F | TCGGTCGGGTATGTACAAGTGG | |
| RLTAA1R | TCTGATGGTTCCGTCAGGGTTAT | |
| RLTAR2F | TCAGGTTTATATCGATGGGGTG | |
| RLTAR2R | TGCTCTTTGAAGCAGTGAAGAG | |
| RLYUCCA1F | AGAACGGTCGGATTCAATAGCAT | |
| RLYUCCA1R | TCAGGGAAGTCCAGGAGGGGTAAC | |
| RLYUCCA2F | TAAAGCCCGTTTTTAACCAGAC | |
| RLYUCCA2R | AAGCCACCGTGATACATACTCC | |
| RLGH3.2F | CGAAGAAGTCATGGCTAAGTGC | |
| RLGH3.2R | CAAACGTACCGTTCCGTACCAC | |
| RLGH3.4F | CCATCACCGAGTTTCTCACAAG | |
| RLGH3.4R | CGTAGACTCCGACTTCACAAATAAG | |
| RLSAUR16F | CTTGATGTTCCCAAAGGCTACTT | |
| RLSAUR16R | GATCTTGAAATGAAGGCTGGTC | |
| RLSAUR76F | CGTGAGCCACCCGCTCTTCCAG | |
| RLSAUR76R | GCGTCGGCGTTCTCCAACATCC | |
| RLIAA6F | TGACCTTGGTTCGAGCAACAGT | |
| RLIAA6R | ACATCTCCGACGAGCATCCAGT | |
| RLIAA8F | TGATGTTGTCGCCGAAAGTTAAG | |
| RLIAA8R | GCCATTGTATTCTTCCGGTATG | |
